# Differing clinical features between Japanese siblings with cerebrotendinous xanthomatosis with a novel compound heterozygous *CYP27A1* mutation: a case report

**DOI:** 10.1186/s12883-022-02711-4

**Published:** 2022-05-25

**Authors:** Shingo Koyama, Yuma Okabe, Yuya Suzuki, Ryosuke Igari, Hiroyasu Sato, Chifumi Iseki, Kazuyo Tanji, Kyoko Suzuki, Yasuyuki Ohta

**Affiliations:** 1 Department of Internal Medicine III, Division of Neurology and Clinical Neuroscience, Yamagata University Faculty of Medicine, 2-2-2 Iida-nishi, Yamagata, 990-9585 Japan; 2 Department of Clinical Neuroscience, Yamagata University Faculty of Medicine, 2-2-2 Iida-nishi, Yamagata, 990-9585 Japan; 3 Department of Psychiatry, Koishikawa Tokyo Hospital, 4-45-16 Otsuka, Bunkyo-ku, Tokyo, 112-0012 Japan; 4 Department of Behavioral Neurology and Cognitive Neuroscience, Tohoku University Graduate School of Medicine, 2-1 Seiryo-machi, Aoba-ku, Sendai, 980-8575 Japan

**Keywords:** CTX, CYP27A1, Cholestanol, Chenodeoxycholic acid, Case report

## Abstract

**Background:**

Cerebrotendinous xanthomatosis (CTX) is an autosomal-recessive lipid storage disorder caused by mutations in the *CYP27A1* gene encoding the key enzyme in the bile acid synthesis, sterol 27-hydroxylase. Here, we report two Japanese CTX siblings with a novel compound heterozygous *CYP27A1* mutation, showing different clinical phenotypes and responses to chenodeoxycholic acid (CDCA) therapy.

**Case presentation:**

The proband, a 32-year-old man, who had chronic diarrhea, bilateral cataracts, and xanthomas, demonstrated progressive neurological manifestations including ataxia, and spastic paraplegia during a 5-year follow-up period despite normalization of serum cholestanol after initiation of CDCA treatment. He also exhibited cognitive decline although improvement had been observed at the beginning of treatment. Follow-up brain magnetic resonance imaging (MRI) revealed pronounced progressive atrophy in the cerebellum, in addition to expanding hyperintense lesions in the dentate nuclei, posterior limb of the internal capsule, cerebral peduncles, and inferior olives on T2-weighted images. In contrast, the two-year-younger sister of the proband presented with chronic diarrhea, cataracts, xanthomas, and intellectual disability but no other neurological symptoms at the time of diagnosis. CDCA treatment lead to improvement of cognitive function and there were no characteristic CTX-related MRI features during the follow-up period. The siblings shared a paternally inherited c.1420C > T mutation (p.Arg474Trp) and a maternally inherited novel c.1176_1177delGA mutation, predicting p.(Glu392Asp*20).

**Conclusions:**

Our cases suggest that early diagnosis and subsequent initiation of CDCA treatment are crucial before the appearance of characteristic MRI findings and severe neurological manifestations related to CTX. Further studies are required to elucidate mechanisms responsible for the clinical diversity of CTX and prognostic factors for long-term outcomes following initiation of CDCA treatment.

**Supplementary Information:**

The online version contains supplementary material available at 10.1186/s12883-022-02711-4.

## Background

Cerebrotendinous xanthomatosis (CTX: OMIM#213,700) is a rare autosomal-recessive lipid storage disease caused by deficiency of the mitochondrial cytochrome P 450 enzyme, sterol 27-hydroxylase (CYP27A1, EC 1.14.15.15) due to mutations in the *CYP27A1* gene [1]. Decreased activity of sterol 27-hydroxylase leads to impaired bile acid synthesis, resulting in reduced production of bile acids, especially chenodeoxycholic acid (CDCA), in addition to elevated serum cholestanol and urine bile alcohols. Clinical manifestations of CTX include neonatal jaundice or cholestasis, refractory diarrhea, juvenile cataracts, tendon xanthomas, osteoporosis, coronary heart disease, and progressive neuropsychiatric disturbances [2–9]. A phenotypic heterogeneity, even within the same family, is a characteristic feature of CTX [2]. Replacement treatment with CDCA can lead to biochemical and clinical improvements [10, 11]. However, once significant neuropsychiatric manifestations are established, clinical symptoms may continue to worsen despite normalization of serum cholestanol levels [3, 8, 12, 13]. Here, we report two Japanese CTX siblings with the identical genotype showing different clinical courses and responses to treatment with CDCA.

## Case presentation

### Case 1

**Fig. 1:**
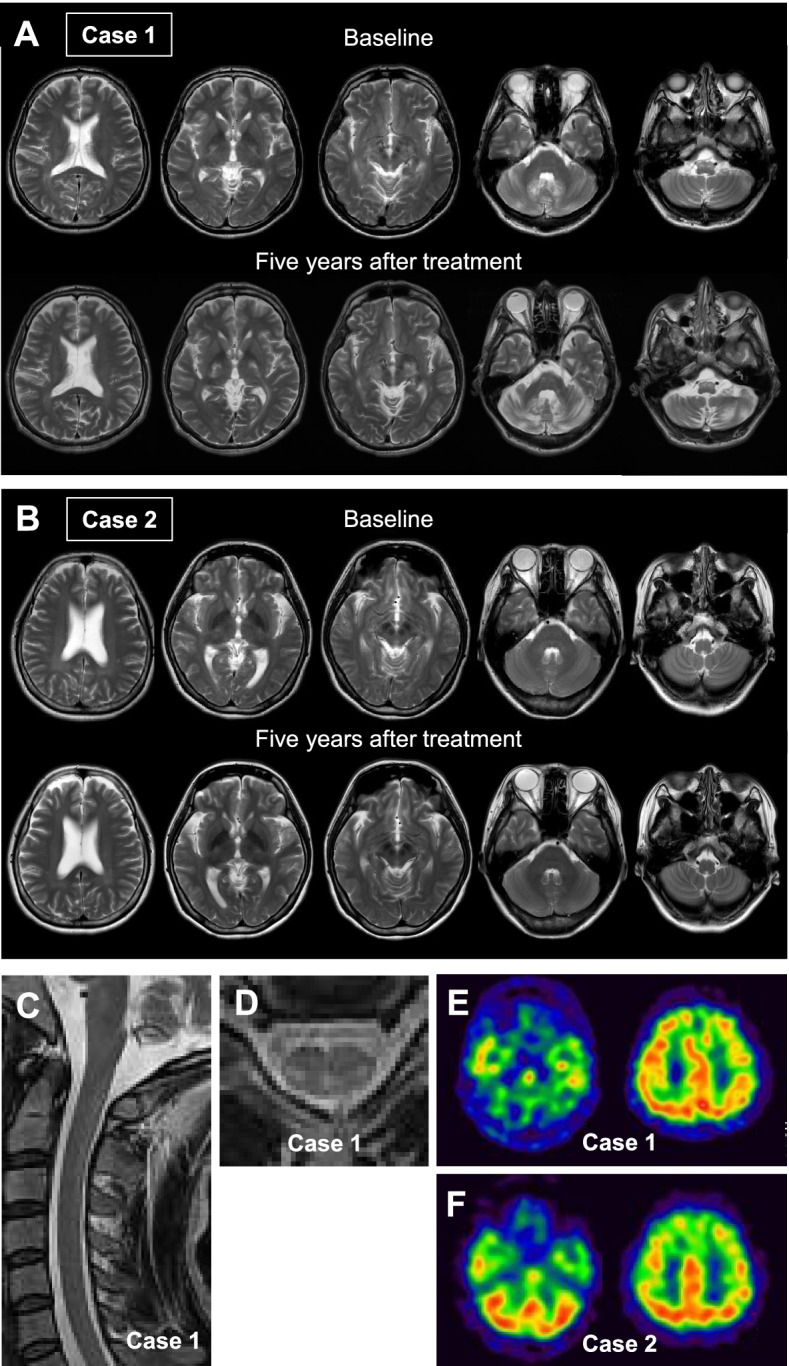
Baseline and 5-year follow-up brain magnetic resonance imaging (MRI) of Case 1 (**A**) and Case 2 (**B**). Axial T2-weighted images at baseline in Case 1 (A, upper panel) and Case 2 (B, upper panel). Five-year follow-up MRI in Case 1 (A, lower panel) and Case 2 (B, lower panel). T2-weighted spinal cord MRI at baseline in Case 1 (**C**, **D**). Cervical cord, sagittal view (**C**). Cervical cord, axial view at level C5 (**D**). Brain ^99^mTc-ethyl cysteinate dimer single photon emission computed tomography in Case 1 (**E**) and Case 2 (**F**)

**Fig. 2:**
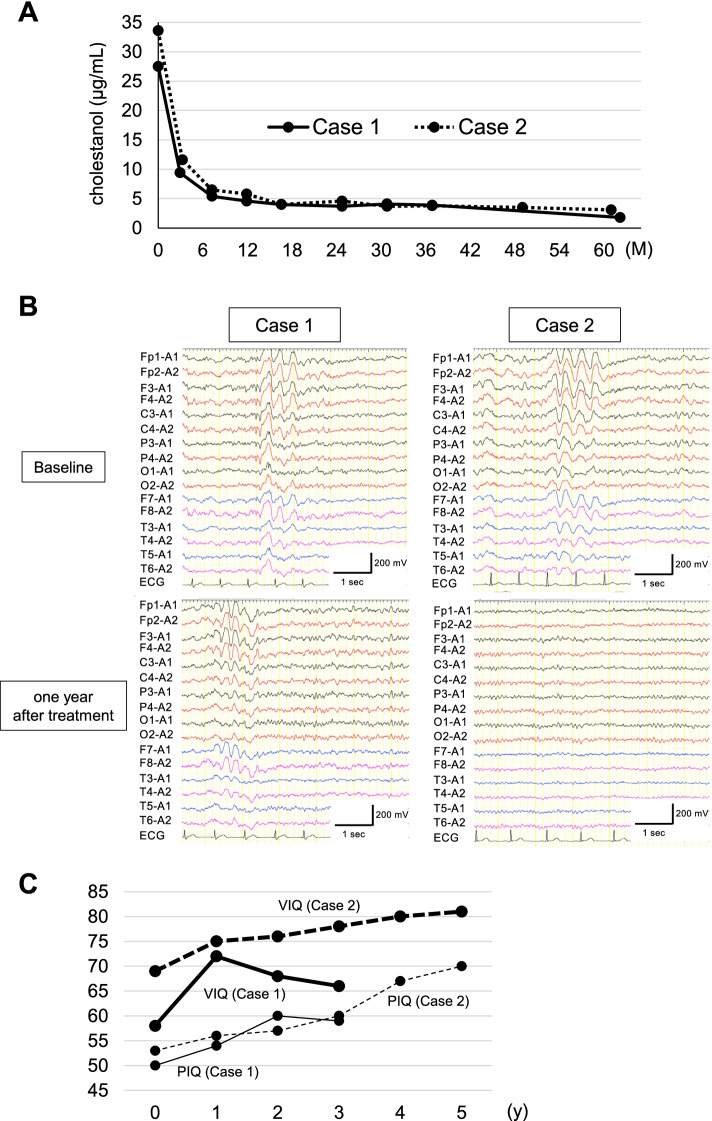
The changes in levels of serum cholestanol during a 5-year follow-up in Case 1 (solid line) and Case 2 (dashed line) (**A**). Electroencephalograms (EEG) of the siblings (**B**). EEG at baseline in Case 1 (B, upper left) and Case 2 (B, upper right). One-year follow-up EEG in Case 1 (B, lower left) and Case 2 (B, lower right). Five-year follow-up of Wechsler Adult Intelligence Scale-III scores (**C**). Thick solid line: verbal IQ (VIQ) in Case 1; thick dashed line: VIQ in Case 2; thin solid line: performance IQ (PIQ) in Case 1; thin dashed line: PIQ in Case 2

The proband, a 32-year-old Japanese man, was the first child of non-consanguineous healthy parents (Supplementary Fig. 1A). There was no past medical history of neonatal jaundice or cholestasis. He had chronic unexplained diarrhea starting within the first year of life. In addition to having learning disabilities, he noted an enlarging tuberous xanthoma on the left knee at the age of 13. At age 31, he underwent bilateral cataract surgery, and developed a gait disturbance, which worsened gradually. At age 32, he visited our hospital. A physical examination revealed a pes cavus deformity of the left foot besides xanthomas of the left knee and bilateral Achilles tendons. On neurological examination, he exhibited impaired smooth pursuit eye movements, dysarthria, ataxia in the trunk and bilateral lower extremities, and spastic paraplegia. Vibration sense in the lower extremities was impaired, while joint position sense was preserved. There was generalized hyperreflexia of all four extremities with bilateral ankle clonus and positive Babinski signs. Parkinsonism was not evident. He had a score of 20/30 on the Mini-Mental State Examination (MMSE). On the Wechsler Adult Intelligence Scale-III (WAIS-III), verbal and performance IQs were 58 and 54, respectively (Fig. 2C). Brain magnetic resonance imaging (MRI) showed hyperintense lesions in the dentate nuclei, posterior limb of the internal capsule, cerebral peduncles, and inferior olives on T2-weighted (Fig. 1A) and fluid-attenuated inversion recovery images. Mild cerebral and cerebellar atrophy was also evident. T2-weighted spinal cord MRI revealed hyperintense lesions localized in the lateral corticospinal tracts and gracile tracts, longitudinally extending from the C1 to Th10 level (Fig. 1C and D). An electroencephalogram (EEG) revealed slow background activity composed of theta and delta waves with bursts of high voltage activity. Epileptic discharges were not present (Fig. 2B). Nerve conduction studies (NCSs) revealed a slightly decreased motor nerve conduction velocity in the posterior tibial nerve, with normal amplitude of compound muscle action potential. Bone mineral density (BMD) at the lumbar spine assessed by dual-energy X-ray absorptiometry was slightly decreased (0.894 g/cm^2^, T-score = -1.3). His serum cholestanol level was elevated to 27.5 µg/mL (normal range, 1.91–3.51 µg/mL) (Fig. 2A).

After obtaining written informed consent, genomic DNA was extracted from the peripheral blood of the patient and his parents. The *CYP27A1* gene was amplified by PCR from the genomic DNA. Sequence analysis identified a compound heterozygous mutation in the proband. One was a novel c.1176_1177delGA mutation in exon 6, resulting in a frame shifting change with a glutamic acid to aspartic acid substitution at position 392 and the new reading frame ending in a stop at position 20, p.(Glu392Asp*20). The other mutation was the known c.1420C > T mutation (p.Arg474Trp) in exon 8. The former mutation was inherited from his mother and the latter from his father (Supplementary Fig. 1B).

The patient was diagnosed with CTX and treated with CDCA at 15 mg/kg daily (750 mg/day), leading to immediate disappearance of the diarrhea. The serum cholestenol concentration gradually decreased in the first year of CDCA treatment and was well-controlled during the follow-up period (Fig. 2A). In EEG after one year of treatment, diffuse slow background activity with bursts of high voltage activity remained evident (Fig. 2B). Despite treatment with CDCA and normalization of serum cholestanol, neuropsychiatric manifestations including dysarthria, dysphagia, and spastic paraplegia continued to worsen. Percutaneous endoscopic gastrostomy was performed due to exacerbating dysphagia. Although an initial improvement in WAIS-III scores was observed with CDCA therapy, scores tended to decrease, particularly for VIQ. However, we could not obtain IQ scores four years after starting therapy because of conversion disorder (Fig. 2C). Follow-up MRI demonstrated progressive signal abnormalities and diffuse brain atrophy especially in the cerebellum (Fig. 1A). Brain ^99^mTc-ethyl cysteinate dimer (^99^mTc-ECD) single photon emission computed tomography (SPECT) revealed marked cerebellar hypoperfusion, as well as mild frontoparietal hypoperfusion (Fig. 1E).

### Case 2

A 30-year-old woman, the younger sister of Case 1, presented to our hospital with suspected CTX. She had been able to attend regular school despite learning difficulties, and had no past medical history of neonatal jaundice or cholestasis. Bilateral juvenile cataracts were diagnosed at the age of 13, and she underwent surgery at the age of 18. At age 20, she had mild diarrhea, while there had been no history of diarrhea during early childhood. A physical examination revealed mild enlargement of both Achilles tendons. A neurological examination found no signs of spasticity, ataxia, or parkinsonism. Neither hyperreflexia nor the extensor plantar response was observed. Her MMSE score was 23/30. Her WAIS-III verbal and performance IQs were 69 and 53, respectively (Fig. 2C). Brain and spinal cord MRI revealed neither obvious signal intensity changes nor atrophy (Fig. 1B). EEG showed slow background activity with bursts of high voltage activity, without epileptic discharges (Fig. 2B). NCS findings were unremarkable. BMD at the lumbar spine measured by DEXA was within the normal range (0.929 g/cm^2^, T-score = -0.7). Her serum cholestanol level was elevated to 33.6 µg/mL (Fig. 2A). Genetic analysis of the *CYP27A1* gene revealed that she was compound heterogeneous for the two above mutations.

She was diagnosed with CTX and treated with CDCA at 15 mg/kg daily, which immediately improved the diarrhea. With treatment, the cholestanol level gradually decreased to within the normal range (Fig. 2A). One year after beginning treatment with CDCA, α-waves were clearly observed on EEG, instead of diffuse slow background activity (Fig. 2B). Her WAIS-III scores improved during the follow-up period (Fig. 2C). Other than a transient obsessive–compulsive disorder, no neurological manifestations were apparent. There was no significant deterioration on brain MRI (Fig. 1B) or in BMD. Brain 99mTc-ECD SPECT revealed mild frontoparietal hypoperfusion, without abnormal cerebellar hypoperfusion (Fig. 1F).

## Discussion and conclusions

*CYP27A1* is the only gene known to be associated with CTX and therefore, the diagnosis is confirmed by the presence of biallelic pathogenic mutations in the *CYP27A1* gene [4, 9, 14]. *CYP27A1* pathogenic mutations include missense and nonsense mutations, splice-site mutations, and insertion/deletion mutations [6, 14]. Regarding the present report, *CYP27A1* gene analysis of family members identified a novel compound heterozygous mutation, c.1176_1177delGA, and the known c.1420C > T mutation. The siblings in this study were enrolled in a nationwide survey in Japan and the c.1176_1177delGA variant was reported in the article by Sekijima et al. as a novel mutation [9]. The c.1176_1177delGA *CYP27A1* mutation is predicted to lead to a frameshift and a premature stop codon. The truncated protein is considered to be functionally null, consistent with the idea that a loss of sterol 27-hydroxylase function mechanism is responsible for CTX. Genetic analysis of the family members revealed that the c.1176_1177delGA mutation is in *trans* with the c.1420C > T mutation, which is known to be pathogenic. The c.1176_1177delGA mutation was not detected in publicly available databases: dbSNP 150 (http://www.ncbi.nlm.nih.gov), 1000 Genomes Project, The Exome Aggregation Consortium (ExAC) (http://exac.broadinstitute.org), and Human Genetic Variation Database (HGVD) (http://www.genome.med.kyoto-u.ac.jp/SnpDB/). Considering these findings, the c.1176_1177delGA mutation was classified as pathogenic according to the recommendation of the American College of Medical Genetics and Genomics and the Association for Molecular Pathology (ACMG-AMP) [15]. Furthermore, this mutation was not listed in the Human Gene Mutation Database (HGMD) (http://www.hgmd.org) or ClinVar (https://www.ncbi.nlm.nih.gov/clinvar/).

Nagai et al. reported a case of triplets with CTX. All three patients equally exhibited xanthomas, cataracts, intellectual disability, pyramidal and cerebellar signs, and sensory loss [16]. A case of three CTX siblings with the similar clinical presentations of tendon xanthomas, cataracts, osteoporosis, mental retardation, cerebellar ataxia, and peripheral neuropathy has also been reported [17]. It has been shown that there are possible associations between c.1421G > A (p.Arg474Gln) and classical form CTX, c.1241G > A (p.Arg405Gln) and spinal form CTX, and c.435G > T (p.Gly145 =) and non-neurological form CTX [9]. On the other hand, striking phenotypic heterogeneity between CTX patients with the same mutation, even within families, has also been reported [2]. In the present siblings, there is considerable phenotypic variability, although they share the same *CYP27A1* mutations. It has been suggested that environmental factors are responsible for phenotypic variability [17]. However, a considerable difference in the severity of intellectual disability and parkinsonism was observed in a pair of identical twins with CTX who had been continuously living together and had similar eating habits [18]. Therefore, it is possible that, rather than environmental factors, currently unknown genetic modifiers or certain epigenetic factors are responsible for the clinical heterogeneity in CTX [18].

After the landmark study published in 1984, in which the long-term efficacy of oral CDCA was demonstrated, it was approved as a first-line treatment for CTX [10]. While CTX has been considered to be a treatable metabolic disorder, clinical deterioration can be observed even after initiation of CDCA treatment [3]. Therefore, it is important to recognize that normalization of serum cholestanol level is not necessarily correlated with a good prognosis [3]. Retrospective cohort studies have demonstrated that the age at diagnosis and initiation of CDCA treatment is associated with the prognosis of CTX patients [8, 12, 13]. It was found that presence of significant neurological manifestations at the time of diagnosis was associated with a poor prognosis in CTX patients who were 25 years of age or older [8]. On MRI, cerebellar vacuolation has been recently indicated as a poor prognostic marker in CTX [19, 20], while absence of dentate nuclei signal changes is associated with a better prognosis [20]. In the present siblings, CDCA treatment lead to a similar gradual decline in serum cholestanol, but the clinical courses were markedly different. The proband (Case 1) showed cognitive decline after beginning treatment with CDCA, despite initial improvement in WAIS-III scores. He also exhibited progressive diffuse brain atrophy and widespread signal changes on follow-up MRI, with progressive neurological manifestations including dysarthria, dysphagia, and spastic paraplegia. On the other hand, the younger sister (Case 2) showed a good response to CDCA treatment, although she had cognitive impairment at the time of diagnosis. Thus, initiation of CDCA treatment before the appearance of characteristic brain MRI findings and severe neurological symptoms seems to be associated with a good clinical course.

In conclusion, we report two CTX siblings with a novel compound heterozygous mutation who showed markedly different phenotypes and clinical courses. Further studies are needed to elucidate mechanisms responsible for the clinical diversity in CTX. There can be a crucial “point of no return” in CTX, after which initiation of treatment cannot prevent progression of the disease and therefore, early diagnosis and treatment are essential [12]. Prognostic factors predicting treatment outcome should be identified for CTX.

## Supplementary Information

**Additional file 1:** **Supplementary Fig. 1.** Pedigree of the family (A). Squares: males; circles: females. Filled symbols indicate affected individuals. Symbols containing black dots represent heterozygous carriers. Arrowhead denotes the proband. Electropherograms of Sanger sequences of the family members (B). Filled bars and arrowheads indicate the c.1176_1177delGA mutation in exon 6 and the c.1420C>T mutation in exon 8, respectively.

## Data Availability

All data supporting our findings are included in the manuscript.

## Abbreviations

CTX: Cerebrotendinous xanthomatosis
CDCA: Chenodeoxycholic acid
MMSE: Mini-Mental State Examination
WAIS-III: Wechsler Adult Intelligence Scale-III
MRI: Magnetic resonance imaging
EEG: Electroencephalogram
NCS: Nerve conduction study
BMD: Bone mineral density
^99^mTc-ECD: ^99^MTc-ethyl cysteinate dimer
SPECT: Single photon emission computed tomography
ExAC: The Exome Aggregation Consortium
HGVD: Human Genetic Variation Database
ACMG-AMP: The American College of Medical Genetics and Genomics and the Association for Molecular Pathology
HGMD: The Human Gene Mutation Database

## References

[1] Cali JJ, Hsieh CL, Francke U, Russell DW (1991). Mutations in the bile acid biosynthetic enzyme sterol 27-hydroxylase underlie cerebrotendinous xanthomatosis. J Biol Chem.

[2] Verrips A, Hoefsloot LH, Steenbergen GC, Theelen JP, Wevers RA, Gabreëls FJ (2000). Clinical and molecular genetic characteristics of patients with cerebrotendinous xanthomatosis. Brain.

[3] Pilo-de-la-Fuente B, Jimenez-Escrig A, Lorenzo JR, Pardo J, Arias M, Ares-Luque A (2011). Cerebrotendinous xanthomatosis in Spain: clinical, prognostic, and genetic survey. Eur J Neurol.

[4] Mignarri A, Gallus GN, Dotti MT, Federico A (2014). A suspicion index for early diagnosis and treatment of cerebrotendinous xanthomatosis. J Inherit Metab Dis.

[5] Nie S, Chen G, Cao X, Zhang Y (2014). Cerebrotendinous xanthomatosis: a comprehensive review of pathogenesis, clinical manifestations, diagnosis, and management. Orphanet J Rare Dis.

[6] Salen G, Steiner RD (2017). Epidemiology, diagnosis, and treatment of cerebrotendinous xanthomatosis (CTX). J Inherit Metab Dis.

[7] Wong JC, Walsh K, Hayden D, Eichler FS (2018). Natural history of neurological abnormalities in cerebrotendinous xanthomatosis. J Inherit Metab Dis.

[8] Duell PB, Salen G, Eichler FS, DeBarber AE, Connor SL, Casaday L (2018). Diagnosis, treatment, and clinical outcomes in 43 cases with cerebrotendinous xanthomatosis. J Clin Lipidol.

[9] Sekijima Y, Koyama S, Yoshinaga T, Koinuma M, Inaba Y (2018). Nationwide survey on cerebrotendinous xanthomatosis in Japan. J Hum Genet.

[10] Berginer VM, Salen G, Shefer S (1984). Long-term treatment of cerebrotendinous xanthomatosis with chenodeoxycholic acid. N Engl J Med.

[11] van Heijst AF, Verrips A, Wevers RA, Cruysberg JR, Renier WO, Tolboom JJ (1998). Treatment and follow-up of children with cerebrotendinous xanthomatosis. Eur J Pediatr.

[12] Yahalom G, Tsabari R, Molshatzki N, Ephraty L, Cohen H, Hassin-Baer S (2013). Neurological outcome in cerebrotendinous xanthomatosis treated with chenodeoxycholic acid: early versus late diagnosis. Clin Neuropharmacol.

[13] Stelten BML, Huidekoper HH, van de Warrenburg BPC, Brilstra EH, Hollak CEM, Haak HR (2019). Long-term treatment effect in cerebrotendinous xanthomatosis depends on age at treatment start. Neurology.

[14] Gallus GN, Dotti MT, Federico A (2006). Clinical and molecular diagnosis of cerebrotendinous xanthomatosis with a review of the mutations in the CYP27A1 gene. Neurol Sci.

[15] Richards S, Aziz N, Bale S, Bick D, Das S, Gastier-Foster J (2015). Standards and guidelines for the interpretation of sequence variants: a joint consensus recommendation of the American college of medical genetics and genomics and the association for molecular pathology. Genet Med.

[16] Nagai Y, Hirano M, Mori T, Takakura Y, Tamai S, Ueno S (1996). Japanese triplets with cerebrotendinous xanthomatosis are homozygous for a mutant gene coding for the sterol 27-hydroxylase (Arg441Trp). Neurology.

[17] Suh S, Kim HK, Park HD, Ki CS, Kim MY, Jin SM (2012). Three siblings with cerebrotendinous xanthomatosis: a novel mutation in the CYP27A1 gene. Eur J Med Genet.

[18] Zádori D, Szpisjak L, Madar L, Varga VE, Csányi B, Bencsik K (2017). Different phenotypes in identical twins with cerebrotendinous xanthomatosis: case series. Neurol Sci.

[19] Mandia D, Besson G, Lamari F, Castelnovo G, Curot J, Duval F (2019). Cholic acid as a treatment for cerebrotendinous xanthomatosis in adults. J Neurol.

[20] Mignarri A, Dotti MT, Federico A, De Stefano N, Battaglini M, Grazzini I (2017). The spectrum of magnetic resonance findings in cerebrotendinous xanthomatosis: redefinition and evidence of new markers of disease progression. J Neurol.

