# Supplementary material for: Differing clinical features between Japanese siblings with cerebrotendinous xanthomatosis with a novel compound heterozygous CYP27A1 mutation: a case report
Source: BMC Neurol. 2022 May 25;22:193. doi: 10.1186/s12883-022-02711-4 (PMC9131546; doi:10.1186/s12883-022-02711-4)
Supplement: Supplementary file 1 — Additional file 1: Supplementary Fig. 1. Pedigree of the family (A). Squares: males; circles: females. Filled symbols indicate affected individuals. Symbols containing black dots represent heterozygous carriers. Arrowhead denotes the proband. Electropherograms of Sanger sequences of the family members (B). Filled bars and arrowheads indicate the c.1176_1177delGA mutation in exon 6 and the c.1420C>T mutation in exon 8, respectively. [file 12883_2022_2711_MOESM1_ESM.pptx]

## Slide 1
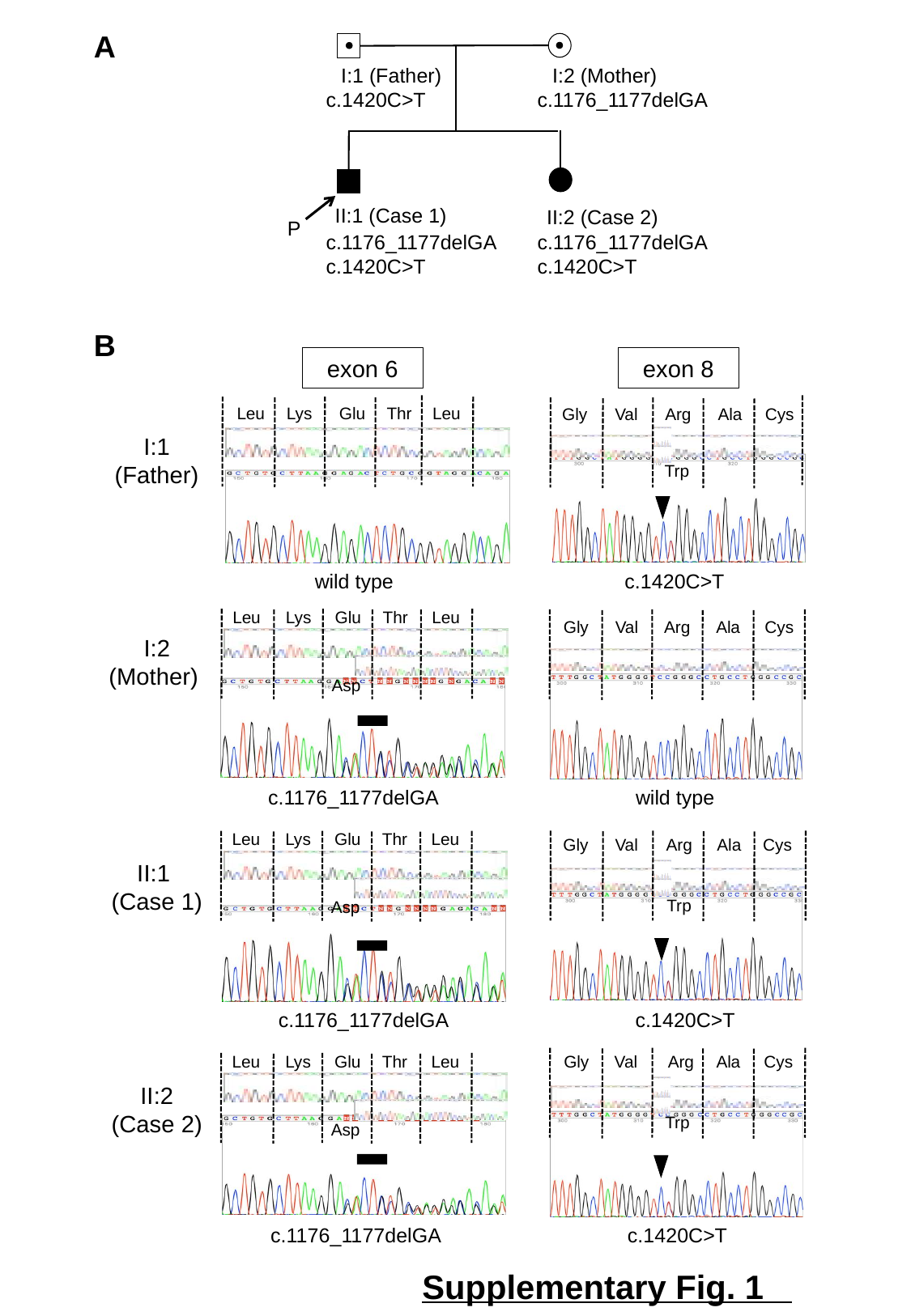

A
I:1 (Father)
I:2 (Mother)
c.1420C>T
c.1176_1177delGA
II:1 (Case 1)
II:2 (Case 2)
P
c.1176_1177delGA
c.1420C>T
c.1176_1177delGA
c.1420C>T
B
exon 6
exon 8
Leu
Lys
Glu
Thr
Leu
Gly
Val
Arg
Ala
Cys
Trp
wild type
c.1420C>T
I:1
(Father)
Leu
Lys
Glu
Thr
Leu
Asp
Ala
Cys
Gly
Val
Arg
c.1176_1177delGA
wild type
I:2
(Mother)
Leu
Lys
Glu
Thr
Leu
Asp
Gly
Val
Arg
Ala
Cys
Trp
c.1176_1177delGA
c.1420C>T
II:1
(Case 1)
Leu
Lys
Glu
Thr
Leu
Asp
Gly
Val
Arg
Ala
Cys
Trp
c.1176_1177delGA
c.1420C>T
II:2
(Case 2)
Supplementary Fig. 1
